# Spontaneous rhythm discrimination in a mammalian vocal learner

**DOI:** 10.1098/rsbl.2022.0316

**Published:** 2022-10-26

**Authors:** Laura Verga, Marlene G. U. Sroka, Mila Varola, Stella Villanueva, Andrea Ravignani

**Affiliations:** ^1^ Comparative Bioacoustics Research Group, Max Planck Institute for Psycholinguistics, Nijmegen, The Netherlands; ^2^ Department of Neuropsychology and Psychopharmacology, Faculty of Psychology and Neuroscience, Maastricht University, Maastricht, The Netherlands; ^3^ Department of Behavioural Biology, University of Münster, Münster, Germany; ^4^ Research Department, Sealcentre Pieterburen, Pieterburen, The Netherlands; ^5^ Center for Music in the Brain, Department of Clinical Medicine, Aarhus University, Aarhus, Denmark

**Keywords:** evolution of rhythm, vocal production learning, auditory perception, rhythm perception, biology of music, harbour seal

## Abstract

Rhythm and vocal production learning are building blocks of human music and speech. Vocal learning has been hypothesized as a prerequisite for rhythmic capacities. Yet, no mammalian vocal learner but humans have shown the capacity to flexibly and spontaneously discriminate rhythmic patterns. Here we tested untrained rhythm discrimination in a mammalian vocal learning species, the harbour seal (*Phoca vitulina*). Twenty wild-born seals were exposed to music-like playbacks of conspecific call sequences varying in basic rhythmic properties. These properties were called length, sequence regularity, and overall tempo. All three features significantly influenced seals' reaction (number of looks and their duration), demonstrating spontaneous rhythm discrimination in a vocal learning mammal. This finding supports the rhythm–vocal learning hypothesis and showcases pinnipeds as promising models for comparative research on rhythmic phylogenies.

## 1. Introduction

The perception of rhythmic sounds is fundamental to human speech and music: upon perceiving a beat, our motor system becomes readily entrained to it. This ability, named beat perception and synchronization [1], is a human universal; yet, its evolutionary route is debated. In our species, rhythm perception relies on the accuracy of the motor system [2,3]. Likewise, vocal production learning (VPL)—the ability we deploy when learning to produce speech sounds—requires precise vocal motor control. One main hypothesis for the evolution of rhythm states that flexible perception of rhythm patterns relies on, and stemmed from, VPL [1,4]. Because humans only constitute one datapoint to test this evolutionary hypothesis of co-occurrence of rhythm and VPL, a cross-species approach is needed to probe whether other VPL species also possess rhythmic abilities.

**Figure 1.**
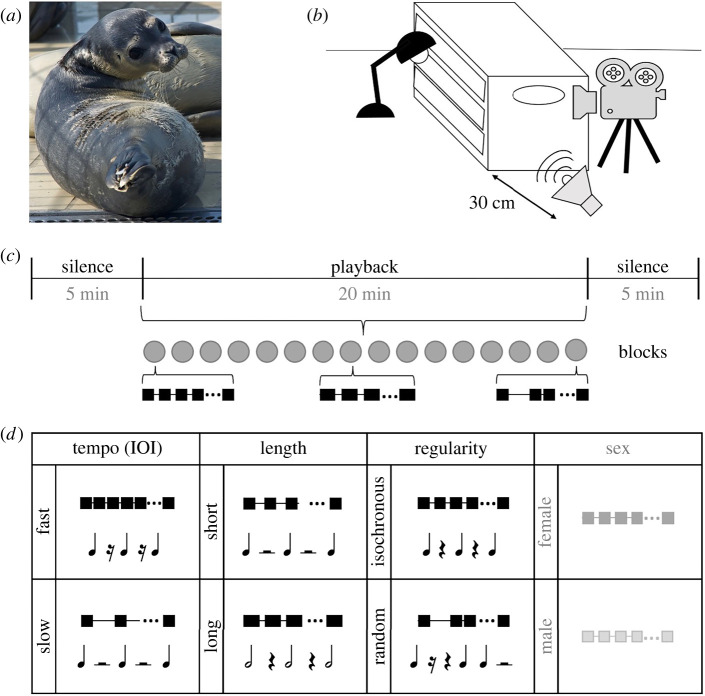
Experimental design. (*a*) Example of the approximate posture shown by a seal turning toward the playback source. (*b*) Tested seals were individually housed in transport boxes, which prevented visual distraction. Playback sounds were broadcasted from the caudal side of the animal and mimicked the sound pressure level of a nearby conspecific. The recording video camera was positioned next to the loudspeaker. Videos were blindly annotated by two raters to quantify the number and duration of head turn towards the playback source. (*c*) Graphic representation of the structure of a playback sequence (*d*) Graphic representation of the three rhythmic factors used in the experiment and their corresponding approximate western music notation. Each square (rectangle when duration is longer) represents a seal pup call.

Surprisingly, to date, no rhythm perception abilities of this kind have been experimentally tested in VPL mammals, while they have been observed—mostly after extensive training—in songbirds [4]. This contrasts with our closest relatives, non-human primates, showing limited VPL and rhythm perception abilities [5–7]. Does any other mammal, apart from humans, have joint rhythm and VPL capacities, as predicted by the vocal learning–rhythm perception hypothesis [1,4]? To answer this question, we identify and test a purported mammalian ‘missing link’ in the evolution of rhythm perception. Because of their advanced VPL abilities, harbour seals (*Phoca vitulina*) are promising candidates to fill this gap and provide comparative evidence for the origins of music and speech in humans [6,8–10]. Do harbour seals' VPL capacities translate into a natural ability to spontaneously discriminate rhythmic patterns, as predicted by the hypothesis [1,4]? Here we test whether untrained, wild-born seals can discriminate music-like rhythmic features in acoustic sequences. To this aim, we exposed 20 infant seals to playbacks of conspecific seal pup calls which differed in their rhythmic properties. Then, similarly to human infant studies, we measured how often and for how long they turned their head toward the sound source in each condition (figure 1). Crucially, the current experiment does not intend to test animals with human music, nor to record their natural rhythmicity; rather, we distil the building blocks of musical rhythm, and we test them in a species-relevant way.

## 2. Methods and results

Our playback experiment tested the ability of 20 harbour seals (11 female, age ≤ 10 months) to discriminate between different rhythmic features (figure 1 and electronic supplementary material [11] for details). A playback sequence consisted of 16 playback blocks (20 min); their order was randomized and they were surrounded by two silent periods (5 min each; total experiment time: 30 min). Each block (duration range: 50–100 s) contained 21 concatenated identical single calls and hence 20 inter-onset intervals (IOIs, i.e. the times between the onset of one call and the onset of the next). Each block simulated three rhythmic factors: (i) tempo: calls were presented at fast (average IOI of 2000 ms) or slow (average IOI 4000 ms) repetition rates; (ii) length: calls' duration could be either short (470–485 ms) or long (945–950 ms); (iii) regularity: IOIs between calls could be isochronous (i.e. metronomic) or random (i.e. arranged to configure different random patterns of temporal intervals). Reference [12] and electronic supplementary material [11] contain details on how these sequences were generated. The sex of the simulated conspecific (50% female calls) was added as a non-rhythmic factor but did not emerge as a significant predictor in any of the statistical models (all *p*-values > 0.10; see electronic supplementary material [11]). Furthermore, the robustness of our results against varying data-cleaning and statistical choices was explored through a multiverse analysis (electronic supplementary material [11]). All factors were based on the natural temporal ranges of seal calls while mimicking the rhythmic essence of music-like sequences (an approximate western music notation is presented as an example in figure 1).

All individuals were kept for clinical reasons only, released immediately after testing, and showed no sign of neurological problems. All procedures involved non-invasive behavioural testing, adhered to current ethical guidelines and were approved by expert veterinarians.

**Figure 2.**
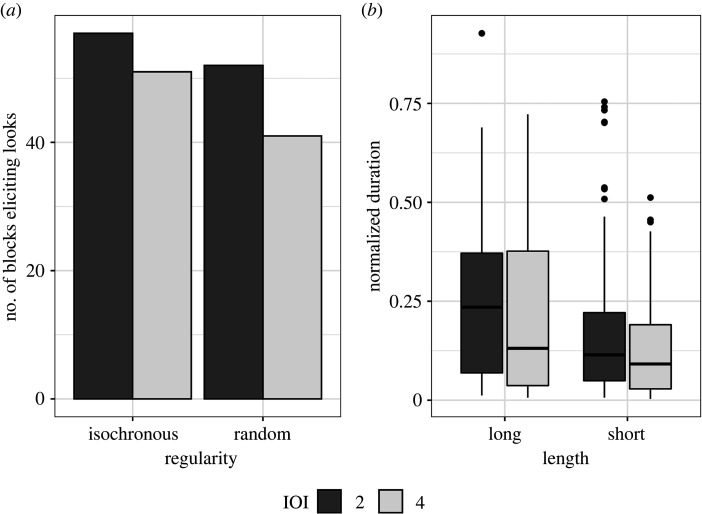
Statistically significant comparisons within rhythmic factors. (*a*) Bar plots depict the number of blocks eliciting at least one look across all seals for tempo and regularity. The number of looks was transformed in a binomial variable (total possible outcomes = 20 seals × 16 blocks = 320). More looks were elicited by faster tempi (IOI2 > IOI4; *p* = 0.022) and by isochronous sequences (isochronous > random; *p* = 0.043), with no significant interaction between the two factors (see electronic supplementary material [11]). (*b*) Boxplot depicting the duration of looks in different conditions; look durations were normalized based on the total duration of each block. Longer looks were elicited by faster tempi (IOI2 > IOI4; *p* = 0.002) and by longer calls (long > short; *p* < 0.001), with no significant interaction. IOI = inter-onset interval. See electronic supplementary material [11] for details.

Our data (figure 2) show that harbour seals, within their first year of life, spontaneously differentiate between rhythmic patterns varying in tempo (i.e. in music, beats per minute), token length (akin to the duration of notes) or regularity (i.e. the predictability of a rhythmic sequence). More specifically, faster tempi (IOI = 2, 0.74 ± 0.07) were more likely to elicit looks as compared to slower tempi (IOI = 4, 0.60 ± 0.08; model estimate −0.62 ± 0.27, bootstrap 95% CI [−1.16;−0.13], *p* = 0.022; binomial generalized mixed model). Isochronous playbacks (0.73 ± 0.07) were more likely to elicit looks compared to random playbacks (0.61 ± 0.08; binomial model estimate −0.54 ± 0.27, bootstrap 95% CI [−1.09; 0.00], *p* = 0.043; figure 2*a*). Concerning look duration, faster tempi elicited significantly longer looks (0.18 ± 0.03) compared to slower tempi (0.12 ± 0.02; model estimate −0.37 ± 0.12, bootstrap 95% CI [−0.69;−0.03], *p* = 0.002). Long calls (0.18 ± 0.03) elicited longer looks than short calls (0.12 ± 0.02; model estimate −0.42 ± 0.12, bootstrap 95% CI [−0.75;−0.09], *p* < 0.001; figure 2*b*). Crucially, the sex of the emitter—the only non-rhythmic variable—did not affect seals' responses (see electronic supplementary material [11]).

## 3. Discussion

Our data show rhythm discrimination abilities in a mammal other than humans. One previous study trained rats to perceive simple, isochronous rhythms: after extensive training, the rats' detection rates, albeit significant, were low and showed little tempo flexibility [13]. Crucially, rats are mammals but not vocal learners. By contrast, with no training and in their infancy, seals were influenced by the three tested rhythmic dimensions while the non-rhythmic one did not elicit a difference in response, suggesting a potential innate ability to discriminate rhythmic patterns. In particular, tempo and length elicited strong responses, while regularity yielded a significant, though smaller, effect which may require further investigation (see also Multiverse analysis, electronic supplementary material [11]).

Our results support the hypothesis that vocal learning species—such as harbour seals—possess developed rhythm perception capacities [1,4,14], and showcase seals' potential as a mammalian model for rhythm evolution research. In addition, our results have ecological implications: they dovetail with the relevance of tempo and patterning of sound production in other pinnipeds [15]. Furthermore, they point toward temporal modulation as building block for vocal rhythmic production in natural contexts, such as mother–offspring recognition, showing another potential parallel to our species [12,16,17].

To reconstruct how humans and other mammals evolved rhythm perception, follow-up comparative research could span four strands, targeting: (1) function, i.e. exploring the socio-ecological value of rhythm in seals versus other species; (2) mechanism, i.e. using electrophysiology to detect neural signatures of rhythm perception; (3) ontogeny, i.e. following the developmental trajectory of rhythmic capacities; (4) phylogeny, i.e. comparing harbour seals to other pinnipeds or non-VPL mammals to ascertain whether rhythm is a matter of common ancestry or convergent evolution.

## Data Availability

Additional analyses, data and scripts supporting the findings highlighted in this article are provided in the electronic supplementary material [11].
